# Social Engagement, Loneliness and Depression Among Older Adults Living Alone: A Systematic Review

**DOI:** 10.1093/geroni/igaf122.3078

**Published:** 2025-12-31

**Authors:** Genevieve Ebulum, Obinna Ezeihuoma

**Affiliations:** David Umahi Federal University of Health Sciences (DUFUHS) Uburu, Ohaozara LGA, Ebonyi state, Nigeria, Enugu, Enugu, Nigeria; University of Pittsburgh, Pittsburgh, Pennsylvania, United States

## Abstract

Loneliness and depression among older adults living alone is a critical public health issue. It is associated with physical and mental health consequences, including increased mortality and morbidity. Social engagement has emerged as a potential intervention to address these issues, yet existing research remains fragmented and lacks comprehensive evaluation. A systematic review was conducted on the role of social engagement in mitigating loneliness and depression among older adults living alone. We reviewed articles published between 2018 and 2024 was conducted, following PRISMA guidelines. Databases including PubMed, CINAHL and PsycINFO were searched using predefined inclusion criteria. The Critical Appraisal Skills Programme (CASP) tool was used to assess the quality of the included studies. Data were extracted and synthesized based on objectives, research design, participants, context, and outcomes. The review included five high-quality studies. Findings revealed that interventions promoting social engagement such as group activities, community programs, and digital platforms significantly reduced loneliness and depression among older adults. However, the effectiveness of these interventions was influenced by factors like cultural norms, resource accessibility, and individual preferences. The review highlights the importance of tailoring social engagement interventions to the specific needs and contexts of older adults. It emphasizes the role of community support, digital inclusion, and policy frameworks in enhancing the reach and effectiveness of these interventions. This systematic review underscores the critical role of social engagement in improving mental well-being among older adults living alone. Evidence-based strategies should prioritize accessibility, inclusivity, and cultural sensitivity to maximize their impact.

